# Vector Approximate Message Passing Based OFDM-IM Detection for Underwater Acoustic Communications

**DOI:** 10.3390/e25121667

**Published:** 2023-12-17

**Authors:** Xiao Feng, Feng Tian, Mingzhang Zhou, Haixin Sun, Zeyad A. H. Qasem

**Affiliations:** 1 School of Communication and Information Engineering, Nanjing University of Posts and Telecommunications, Nanjing 210003, China; fengxiao@njupt.edu.cn; 2 Key Laboratory of Southeast Coast Marine Information Intelligent Perception and Application, Ministry of Natural Resources, Zhangzhou 363099, China; hxsun@xmu.edu.cn; 3 School of Informatics, Xiamen University, Xiamen 361005, China; 4 School of Electronic and Computer Engineering, Peking University, Shenzhen 518005, China

**Keywords:** underwater acoustic communications, OFDM-IM, data detection, vector approximate message passing

## Abstract

Orthogonal frequency division multiplexing with index modulation (OFDM-IM) has great potential for the implementation of high spectral-efficiency underwater acoustic (UWA) communications. However, general receivers consisting of the optimal maximum likelihood detection suffer from high computational load, which prohibits real-time data transmissions in underwater scenarios. In this paper, we propose a detection based on a vector approximate message passing (VAMP) algorithm for UWA OFDM-IM communications. Firstly, a VAMP framework with a non-loopy factor graph for index detection is formulated. Secondly, by utilizing the sparsity inherently existing in OFDM-IM symbols, a novel shrinkage function is derived based on the minimum mean square error criterion, which guarantees better posterior estimation. To reduce the errors from estimated non-existing indices, one trick is utilized to search the elements from the look-up table with the minimal Euclidean distance for the replacement of erroneously estimated indices. Experiments verify the advantages of the proposed detector in terms of low complexity, robustness and effectiveness compared with the state-of-art benchmarks.

## 1. Introduction

Underwater acoustic (UWA) communications are dominant technologies for data exchanges in broad sea areas, which have been successfully used to accomplish underwater tasks such as marine environmental monitoring, marine security surveillance and resource exploration. As the requirements for underwater network deployments and large-volume marine data acquisition increase, multicarrier UWA communications have attracted the attention of researchers due to high spectral efficiency (SE), where Orthogonal Frequency Division Multiplexing (OFDM) and index modulation with OFDM (OFDM-IM) are the outstanding and representative schemes. OFDM-IM has the robustness to multipath fading of UWA channels and exploits the idle space resources as compensation for the limited frequency bandwidth. OFDM-IM communications are highly desired to be further studied for emerging underwater applications.

The concept of index modulation (IM) originates from spatial modulation (SM) in multiple-input multiple-output (MIMO) systems. The difference between OFDM-IM from SM-MIMO is that OFDM-IM employs the indices of subcarriers in the frequency domain instead of the indices of antennas [1]. IM separates the data into two parts, i.e., the constellation data and the index data, hence OFDM-IM conveys useful information using both constellation symbols and subcarrier indices. OFDM-IM is flexible to achieve the tradeoff between bit error rate (BER) performance and SE by designing different subcarrier activation patterns (SAPs), which determine the activated or inactivated subcarriers. In OFDM-IM, additional information requires special handling because the conventional detection for constellation symbols is only part of data detection modules. The detection of SAPs is the prerequisite for constellation data detection, which is the key to avoiding decoding error propagation. For OFDM-IM detection, maximum likelihood (ML) detection is the best, which searches the optimal indices and constellation points among all combinations. The complexity of ML detection increases exponentially with the number of index combinations as well as the order of constellation modulations. Even the searches have been employed for each subblock, the complexity is heavy, especially for the multicarrier systems with large-number subcarriers and high-order constellation modulations. A log-likelihood (LLR) detector can achieve near-optimal ML performance with reduced complexity [1]. If the ratio of activated subcarriers is low, the LLR detector tends to find nonexistent index combinations with high probability. Subsequently, many sub-optimal detectors are developed [2,3,4]. The work in [3] proposes a low-complexity detector that conveys data using all possible SAPs to avoid errors from invalid SAPs. The mapping of non-fixed length bits makes the communication system complicated. The work in [4] proposes a modified k-largest-value (klv) detector, which chooses *k* active indices with the largest values according to the active likelihood metrics. This method provides a method for dealing with illegal SAPs and operates on each subcarrier.

As research deepens, the inherent characteristics of OFDM-IM symbols from IM concept and SAPs are further exploited. SAPs are constructed by zero subcarriers and activated subcarriers, which makes OFDM-IM symbols present inherent sparsity characteristics. The sparsity leverages powerful tools to solve the detection problem. The paper [5] proposes to interpret data detection as a problem of convex optimization, on which the AP constraints are imposed, and then a semi-definite relaxation method is utilized. The convergence speed of this method is severely limited and the error floor exists in high SNR ranges. Exploiting the sparsity of symbols, compressed sensing (CS) based methods have recently emerged. The work [6] proposes a CS-assisted signaling strategy, based on which an iterative residual check (IRC)-based detector is formulated. An AMP-based detector is proposed for OFDM-IM by exploiting the statistic of OFDM-IM symbols in the frequency domain [7]. The work [8] proposes to use the AMP framework to realize iterative channel estimation and data detection for OFDM-IM. Approximate Message Passing (AMP) algorithm belongs to the Bayesian estimation framework [9,10]. The simplifications of the Gaussian message according to the central limit theorem and Taylor expansions make the IM detector efficient depending on several posterior parameters. One constraint of the AMP method is the sensitivity to the non-Gaussianity of the dictionary matrix. The generalized AMP (GAMP) is incorporated as a step for joint phase-noise Estimation and decoding in OFDM-IM [11]. The performance of these OFDM-IM detectors for UWA communications cannot be concluded and is to be verified and analyzed.

OFDM-IM was first introduced to UWA communications in [12]. The work verifies the reduced PAPR effects due to power averaging by the inactivated subcarriers and better BER performance compared with OFDM. Since then, OFDM-IM-based UWA communications have been developing. Combining the IM with advanced OFDM schemes, many UWA OFDM-IM transceivers are proposed such as the fully quadrature subcarrier-index shift keying OFDM (FQSISK-OFDM) modulation scheme [13], which put their emphasis on the design of activation strategy. The work [14] combines the IM concept with Orthogonal Time Frequency Space (OTFS) and proposes a Hamming distance optimized model to modify the index combinations. Currently, there is a lack of a design scheme for a low-complexity OFDM-IM detector for UWA communications and a detailed analysis of the effects from the UWA channel for UWA OFDM-IM systems. To fill this gap, this paper considers the OFDM-IM receiver design and especially focuses on data detection, which is a major challenge for real-time and high SE data transmission schemes in the UWA physical layer.

In this paper, we involve the data detection for OFDM-IM in the Bayesian estimation framework. Instead of a loopy factor graph (FG), a vector approximate message passing (VAMP) detector based on a scalar FG is proposed. An appropriate statistical prior model is beneficial for the achievement of the optimal Bayesian solution. By exploiting the sparsity of symbols in the frequency domain, the SAP constraint is considered using the statistical prior and a novel minimum mean square error (MMSE)-optimal shrinkage function is derived. The data reconstruction performance is improved through the forward-back message passing scheduling, where the involved statistical parameters are learned automatically. Inherently from the robustness of VAMP for the deviations of Gaussian matrices [15], the proposed detector is less sensitive to the non-Gaussianity of the measurement matrix which is composed of unknown channel components and presents good generality and convergence. Aiming to mitigate the possible invalid SAPs, a modified trick using the criterion of minimal Euclidean distance with the space of a look-up table is used to replace the possible non-existent results. The VAMP-based detection has relatively low complexity, which is advantageous for real-time data transmission. Simulation results verify the proposed receiver outperforms the benchmarks in terms of complexity, BER as well as robustness to the time-varying UWA channel.

The rest of this paper is organized as follows. Section 2 introduces the UWA OFDM-IM communication systems. The proposed VAMP-based detector is presented in Section 3. The computation complexity and numerical experiments are analyzed in Section 4. Section 5 makes the conclusive remarks.

Notations: Lower case boldface letter x and upper case boldface letter X denote vector and matrix, respectively. ${\left(\cdot \right)}^{T}$ and ${\left(\cdot \right)}^{H}$ stand for the transpose and conjugate transpose operation. $\mathrm{diag}\left\{x\right\}$ is the diagonalized operation with x as the diagonal element. $E\left\{\cdot \right\}$ and $\mathrm{var}\left\{\cdot \right\}$ denote the expectation and variance operation. $\left\lfloor \cdot \right\rfloor$ denotes the floor operation.

## 2. System Model

**Figure 1:**
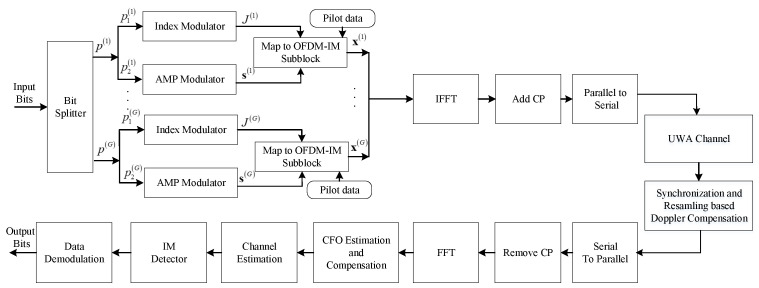
OFDM-IM system for UWA communications.

In this paper, the OFDM-IM-based UWA communication system is considered. The system framework is shown in Figure 1. There are a total *B* bits to be transmitted and input to the bit splitter. The system has *N* subcarriers, and these subcarriers are split into *G* groups. Each group includes n=NNgG subcarriers. Correspondingly, the bits are also split as *G* groups, and there are *p* bits in each group. The *p* bits include two parts, i.e., $p_{1}$ bits for index selection and $p_{2}$ bits for amplitude and phase modulation (APM). $p_{1}$ bits are mapped as the indices of *k* activated subcarriers, which are extracted from the index combinations in a look-up table. $p_{1}$ is determined by $\left\lfloor \log_{2}\left(\begin{array}{c} n\\ k \end{array}\right)\right\rfloor$ which is the logarithm of the index combination number. The total number of data subcarriers is $N_{d}=kG$. Considering the *g*-th group of the OFDM-IM subblock, the indices of activate subcarriers in the look-up table J are given by

(1) $$J^{\left(g\right)}=\left\{j_{1}^{\left(g\right)},j_{2}^{\left(g\right)},\cdots ,j_{k}^{\left(g\right)}\right\},$$

where $j_{\gamma }^{\left(g\right)}\in \left\{1,\cdots ,n\right\}$ for γ=1,⋯,k and g=1,⋯,G. The elements of $J^{\left(g\right)}$ are arranged in an ascending order. The size of Table J is $\left|J\right|=2^{p_{1}}$. $p_{2}$ bits are mapped as *M*-ary constellation points from the constellation alphabet S. $p_{2}=k\log_{2}\left(M\right)$ where $M=\left|S\right|$ is the modulation order. Mapping symbol $s^{\left(g\right)}={\left\{s_{\gamma }^{\left(g\right)}\right\}}_{\gamma =1}^{k}$ are appended on the *k* activated subcarriers with normalized power, i.e., $E\left\{s^{H}s\right\}=1$.

A coherent UWA communication system is required to track the channel effects. Besides data subcarriers, there are $N_{p}$ subcarriers allocated as comb pilots for the tracking of time-varying UWA channels. The information of pilot subcarriers is perfectly known. The remaining $N_{u}=N-N_{p}-N_{d}$ subcarriers are idle. The *g*th OFDM subblock can be expressed as $x^{\left(g\right)}={\left[x_{1}^{\left(g\right)},x_{2}^{\left(g\right)},\cdots ,x_{n}^{\left(g\right)}\right]}^{T}$. Concatenate *G* OFDM-IM subblocks, and OFDM-IM data frame is given by

(2) $$x={\left[{\left(x^{\left(1\right)}\right)}^{T},{\left(x^{\left(2\right)}\right)}^{T},\cdots {\left(x^{\left(G\right)}\right)}^{T}\right]}^{T},$$

where $x\in C^{N\times 1}$ denotes the symbols to be modulated on *N* subcarriers. The *m*th subcarrier is with the frequency $f_{m}=f_{c}+m\Delta f$, where Δf denotes the subcarrier spacing. The symbol duration is $T=\frac{1}{\Delta f}$. Before transmission, the OFDM-IM is transformed as the time-domain signal through the inverse fast Fourier transform (FFT) operation, then the $N_{cp}$-length cyclic prefix (CP) is appended to mitigate the inter-symbol interference (ISI), and the time duration of CP $T_{cp}$ is larger than the maximum path delay. The SE is given by

(3) $$\eta =\frac{G\left(\left\lfloor \log_{2}\left(\begin{array}{c} n\\ k \end{array}\right)\right\rfloor +k\log_{2}\left(M\right)\right)}{N+N_{cp}}\left(bit/s/Hz\right).$$

The baseband signal is upshifted to the passband given by

(4) $$\tilde{x}\left(t\right)=2ℜ\left\{\left[\sum_{m=1}^{N}x\left[m\right]e^{j2\pi m\Delta ft}g\left(t\right)\right]e^{j2\pi f_{c}t}\right\},t\in \left[-T_{cp},T\right],$$

where $g\left(t\right)$ is the pulse shaping filter, and $g\left(t\right)=1$ when $t\in \left[-T_{cp},T\right]$, otherwise $g\left(t\right)=0$.

As shown in Figure 1, the signal passes through the UWA channel. The UWA channel is generally expressed as a time-varying model given by

(5) $$h\left(\tau ,t\right)=\sum_{l=1}^{L}\alpha_{l}\left(t\right)\delta \left(\tau -\tau_{l}\left(t\right)\right),$$

where *L* is the total number of paths, $\alpha_{l}\left(t\right)$ and $\tau_{l}\left(t\right)$ denote the path amplitude and path delay for the *l*th path. Assuming all paths have the same Doppler scaling factor *a*, then $\tau_{l}\left(t\right)=\tau_{l}-at$ [16]. After passing through the channel, the received signal is given by

(6) $$\tilde{y}\left(t\right)=\sum_{l=1}^{L}\alpha_{l}\tilde{x}\left(\left(1+a\right)t-\tau_{l}\right)+\tilde{v}\left(t\right),$$

where $\tilde{v}\left(t\right)$ is the passband additive white Gaussian noise (AWGN). Due to the Doppler effects, the received signal suffers from compressing and broadening effects. The scalar coefficient *a* is estimated by the resampling method as $\hat{a}=\frac{{\tilde{T}}_{rx}}{{\tilde{T}}_{tx}}-1$, where ${\tilde{T}}_{tx}$ is the length of the transmitted signal and the length of the received signal ${\tilde{T}}_{rx}$ is calculated by cross-correlating the linear frequency modulated (LFM) preambles of neighboring frames. After the resampling and downshifting operation, the baseband received signal is given by

(7) yt=LPFy˜tt1+a^1+a^e−j2πfct≈ej2πϵt∑mxmej2πmΔft∑lαle−j2πfmτlgt−τl+vt,

where $v\left(t\right)$ denotes the baseband AWGN and $\epsilon =\frac{a-\hat{a}}{1+\hat{a}}f_{c}$ is the residual carrier frequency offset (CFO). To discretize the baseband signal $y\left(t\right)$ at the baseband rate 11BB, then the *p*th received sample is given by $y\left[p\right]=e^{\frac{j2\pi \epsilon p}{B}}\sum_{m}\left\{x\left[m\right]e^{\frac{j2\pi mp}{N}}\sum_{l}\alpha_{l}e^{-j2\pi f_{m}\tau_{l}}g\left(p-l\right)\right\}+v\left[p\right]$. Through expansion and union simplification operation, the received samples are denoted as

(8) $$y\left[p\right]=e^{\frac{j2\pi \epsilon p}{B}}\sum_{l}h_{l}\sum_{m}x\left[m\right]e^{\frac{j2\pi m\left(p-l\right)}{N}}g\left[p-l\right]+v\left[p\right],$$

where hl=∑m=−NN22KK22−1Cfmej2mlN and $C\left(f\right)=\sum_{p}\alpha_{p}e^{-j2\pi f\tau_{p}}$ is the channel frequency response.

Based on Formula (8), CFO matrix is defined as $\Psi \left(\epsilon \right)=\mathrm{diag}\left(1,e^{\frac{j2\pi \epsilon }{B}},\cdots ,e^{\frac{j2\pi \epsilon \left(N-1\right)}{B}}\right)$. Part of inactive subcarriers is used for CFO mitigation. The process is operated in the frequency domain based on the goal of minimizing the leaked energy of the zero subcarriers after the signal passes through the channel. After CP removal and FFT transform, the received signal in the frequency domain is denoted as Y=Fy, where F is the Discrete Fourier Transform (DFT) matrix and y is the received signal in the time domain. The selection matrix Ξ extracts zero subcarriers from Y, and the energy is expressed as ${∥\Xi \mathrm{Fy}∥}_{2}^{2}$. Assuming CFO is perfectly known and compensated, the optimization problem is formulated as

(9) $$\hat{\epsilon }=\underset{\epsilon }{\arg\min}{∥\Xi F\Psi \left(\epsilon \right)y∥}_{2}^{2}.$$

One dimensional (1-D) search method is used to solve the problem (9) [17]. The detailed effects of CFOs are analyzed in the following experiments. With $\hat{\epsilon }$, the received signal components are corrected through phase reversal. Then the inter-carrier interference (ICI) is mitigated and the ICI-free signal is obtained.

Channel state information (CSI) h is obtained through the channel estimation module. $N_{p}$ pilot subcarriers are utilized as input, and the received pilot subcarriers are the output, then the channel estimation problem can be formed as a sparse signal recovery (SSR) problem. To design efficient channel estimation, representative CS-based methods can be involved.

Then frequency-domain channel H is fed to the data detection module. For the detection of OFDM-IM, symbol detection includes index detection and constellation symbol detection. To realize reliable data detection, the signal for the *g*th group $y^{\left(g\right)}$ in Y in the frequency domain is expressed as

(10) $$y^{\left(g\right)}=H^{\left(g\right)}x^{\left(g\right)}+w^{\left(g\right)},g=1,2,\cdots ,G,$$

where $H^{\left(g\right)}\in C^{n\times n}$ is a diagonal matrix with the components from the *g*th group vector in H and $w^{\left(g\right)}$ is the *g*th group Gaussian noise vector. ML detection is the optimal method and searches all the combinations of indices and constellation points, which is generally defined as

(11) $$\left({\hat{J}}^{\left(g\right)},{\hat{s}}^{\left(g\right)}\right)=\underset{J^{\left(g\right)},s^{\left(g\right)}}{\arg\min}\sum_{\gamma =1}^{k}{\left|y_{j_{\gamma }^{\left(g\right)}}^{\left(g\right)}-h_{j_{\gamma }^{\left(g\right)}}^{\left(g\right)}s_{\gamma }^{\left(g\right)}\right|}^{2},$$

where $y_{\chi }^{\left(g\right)}$ and $h_{\chi }^{\left(g\right)}$, χ=1,2,⋯,n, are the corresponding signal element and channel coefficient of the *g*th OFDM-IM subblock. ML detector achieves the optimal error performance. However, it has high complexity which exponentially increases with the size of subblocks and the modulation order. It is impractical for cases with a large number of subcarriers or high-order modulation in UWA communications. Therefore, it is desired to design a low-complexity and effective detector for UWA communications. In the following contents, we propose a novel detector based on VAMP theory by exploiting the inherent sparsity of OFDM-IM symbols in the frequency domain.

## 3. Proposed Method

OFDM-IM symbols with different non-zero supports exhibit sparse structure, which is different from the OFDM frame. In the OFDM frame, almost all subcarriers are required for data transmission. To solve the problem of data detection in OFDM-IM, VAMP based framework is incorporated by considering the symbol sparsity generated from the SAPs. In this section, the concept of VAMP detection and detailed message scheduling is first introduced. Then the VAMP-based data detection with the designed prior aided shrinkage functions is presented.

### 3.1. VAMP Framework for IM Detection

According to the Equation (10), the data detection problem is defined as

(12) $${\hat{x}}^{\left(g\right)}=\underset{x^{\left(g\right)}\in S}{\arg\min}\left\{{∥y^{\left(g\right)}-H^{\left(g\right)}x^{\left(g\right)}∥}_{2}^{2}\right\}.$$

The problem means to recover the vector $x^{\left(g\right)}$ from noisy linear observation $y^{\left(g\right)}$ with noise $w^{\left(g\right)}$. $w^{\left(g\right)}$ follows Gaussian distribution with the element $w^{\left(g\right)}∼\mathrm{CN}\left(0,\gamma_{w}^{-1}\right)$, and $\gamma_{w}$ is the noise precision. Once the problem is solved, the indices are detected and the symbols are equalized jointly. The detection is executed for each group, and the superscript *g* is omitted for simplification.

In the field of digital communications, many problems such as channel estimation, data detection or user detection, are involved as Bayesian estimation problems. The key to the Bayesian message-passing graph is appropriate assumptions about the prior distribution and Gaussian noise. The standard linear regression problem corresponding to the problem in (12) is given by

(13) $$\hat{x}=\underset{x}{\arg\min}\frac{1}{2}{∥y-Hx∥}_{2}^{2}+f\left(x\right),$$

where $f\left(x\right)$ is the penalty function. $y\in C^{\tilde{M}\times 1}$ and $H\in C^{\tilde{M}\times \tilde{N}}$ is the measurement matrix, which is the diagonal matrix with the channel components. Relate the measurement matrix H with the system model in Section 2, $\tilde{M}=\tilde{N}=n$.

VAMP is first proposed in [15] to solve the problem (13), which has been proved robust to a broader class of large random matrices Φ compared with conventional AMP method [9]. Assuming known prior function $p\left(x\right)$ and likelihood function $p\left(y|x\right)$, the posterior function is calculated through Bayesian rule as

(14) $$p\left(x|y\right)=\frac{p\left(y|x\right)p\left(x\right)}{p\left(y\right)},$$

where $p\left(y\right)=\int p\left(y|x\right)p\left(x\right)dx$. According to different estimate criteria, the minimum mean square (MMSE) estimation is ${\hat{x}}_{\mathrm{mmse}}=\underset{\tilde{x}}{\arg\min}\int ∥x-\tilde{x}∥p\left(x|y\right)dx=E\left[x|y\right]$ and the maximum a posteriori (MAP) estimation is ${\hat{x}}_{\mathrm{map}}=\underset{x}{\arg\max}p\left(x|y\right)$.

The basic VAMP framework is given as in Algorithm 1. In Algorithm 1, $\eta \left(\cdot \right)$ is the denoising function which is parameterized by r and γ. $\langle \eta^{\prime }\left(r_{k},\gamma_{k}\right)\rangle$ is the divergence at $r_{k}$ and $\eta^{{}^{\prime }}\left(r_{k},\gamma_{k}\right)=\mathrm{diag}\left[\frac{\partial \eta \left(r_{k},\gamma_{k}\right)}{\partial r_{k}}\right]$. The Onsager term $\alpha_{k}r_{k}$ cancels the correlation between Φ and x and guarantees the Gaussianity of estimated errors.
**Algorithm 1** Vector AMP (SVD Version)**Input:** Measurement $y\in C^{\tilde{M}\times 1}$, dictionary matrix $H\in C^{\tilde{M}\times \tilde{N}}$, maximum iteration number *T*, denoising function $\eta \left(\cdot \right)$, noise precision $\gamma_{w}$**Output:** Recovered channel vector ${\hat{x}}_{T}$  1:Initialization: t=0, $r_{0}$, $\gamma_{0}\geq 0$  2:Compute SVD of $\left[U,S,V\right]=\mathrm{svd}\left(H\right)$, $U^{H}U=I$ and $V^{H}V=I$, $s=\mathrm{diag}\left\{S\right\}$, R=rank(Φ)  3:Compute $\tilde{y}=S^{-1}Uy$  4:**while** t<T **do**  5:      ${\hat{x}}_{k}=\eta \left(r_{k},\gamma_{k}\right)$  6:      $\alpha_{k}=\langle \eta^{\prime }\left(r_{k},\gamma_{k}\right)\rangle$  7:      r˜k=x^k−αkrkx^k−αkrk1−αk1−αk  8:      γ˜k=γk1−αkγk1−αkαkαk  9:      $d_{k}=\gamma_{w}\mathrm{diag}{\left\{\gamma_{w}s^{2}+{\tilde{\gamma }}_{k}1\right\}}^{-1}s^{2}$10:      γk+1=γ˜kdkγ˜kdkNR−dkNR−dk11:      rk+1=r˜k+NRVdiagdkdkdkdky˜−VHr˜k12:**end while**

**Figure 2:**
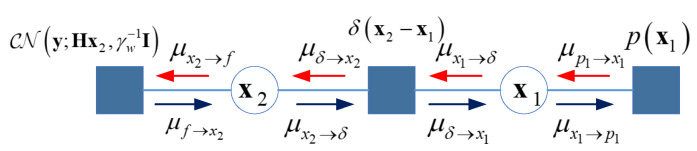
Factor graph of VAMP based detection.

To involve the problem (12) into the VAMP framework, the factor graph and message scheduling of the VAMP-based detection are shown in Figure 2.

In Figure 2, the leftmost node denotes the likelihood function defined as $p\left(y;Hx_{2},{\gamma_{w}}^{-1}I\right)=\prod_{m=1}^{n}\left(\frac{\gamma_{w}}{2\pi }\right)\exp\left(-\frac{\gamma_{w}{∥y_{m}-H_{m}^{T}x_{2}∥}_{2}^{2}}{2}\right)$, where $H_{m}^{T}$ is the *m*th row of the measurement matrix H. The rightmost factor node denotes the prior distribution $p\left(x_{1}\right)$. The original variable node x is divided as $x_{1}$ and $x_{2}$, which are connected by the Dirac delta function $\delta \left(x_{2}-x_{1}\right)$. Then the joint probability distribution is $p\left(y,x_{2},x_{1}\right)=p\left(y|x_{2}\right)\delta \left(x_{2}-x_{1}\right)p\left(x_{1}\right)$. The message passing scheduling in the non-loopy graph includes three parts:

- Approximate belief

The expectation propagation (EP) is adopted for belief calculation using the Gaussian approximation and moment matching. For each variable node, the marginal function is the product of all impinged messages. We denote the approximate message for $x_{i}$ as $\mathrm{CN}\left(x_{i};{\hat{x}}_{i},\beta_{i}^{-1}I\right)$, where i=1,2, i.e., $b_{sp}\left(x_{2}\right)=\mu_{f\to x_{2}}\left(x_{2}\right)\mu_{\delta \to x_{2}}\left(x_{2}\right)$ and $b_{sp}\left(x_{1}\right)=\mu_{p\to x_{1}}\left(x_{1}\right)\mu_{\delta \to x_{1}}\left(x_{1}\right)$. The approximate belief $b_{app}\left(x_{i}\right)$ for the node $x_{i}$ is determined by expectation ${\hat{x}}_{i}$ and covariance $\beta_{i}^{-1}$ with respect to the marginal function $b_{sp}\left(x_{i}\right)$, i.e., $E\left[x_{i}|b_{sp}\left(x_{i}\right)\right]$ and $\langle \mathrm{diag}\left(\mathrm{cov}\left[x_{i}|b_{sp}\left(x_{i}\right)\right]\right)\rangle$.

- Message from variable node to factor node

The message from the variable node to the factor node is $\mu_{x_{i}\to f}\left(x_{i}\right)\propto \frac{b_{app}\left(x_{i}\right)}{\mu_{f\to x_{i}}\left(x_{i}\right)}$. Because all beliefs are approximated as the Gaussian messages, the messages at *t*th iteration are assumed as $\mu_{\delta \to x_{2}}\left(x_{2}\right)∼\mathrm{CN}\left(r_{2t},\gamma_{2t}^{-1}I\right)$ and $\mu_{\delta \to x_{1}}\left(x_{1}\right)∼\mathrm{CN}\left(r_{1t},\gamma_{1t}^{-1}I\right)$.

- Message from factor node to variable node

Message from factor node to variable node is μf→xixi∝∫∼xifxi∏xj∈Nfxj∈Nfxixiμxj→fxj, where $\int_{∼x}$ is to integrate over the other connected factors except x. $N\left(f\right)$ is the neighboring node of *f*.

According to the sum-product rules, the bidirectional messages for each edge are derived as $\mu_{f\to x_{2}}\left(x_{2}\right)=f\left(x_{2}\right)$, $\mu_{p1\to x_{1}}\left(x_{1}\right)=p\left(x_{1}\right)$, $\mu_{\delta \to x_{2}}\left(x_{2}\right)=\int \delta \left(x_{2}-x_{1}\right)\mu_{x_{1}\to \delta }\left(x_{1}\right)dx_{1}$ and $\mu_{\delta \to x_{1}}\left(x_{1}\right)=\int \delta \left(x_{2}-x_{1}\right)\mu_{x_{2}\to \delta }\left(x_{2}\right)dx_{2}$. Due to the equivalent relation in $\delta \left(x_{2}-x_{1}\right)$, $\mu_{x_{1}\to \delta }\left(x_{1}\right)=\mu_{\delta \to x_{2}}\left(x_{2}\right)$ and $\mu_{x_{2}\to \delta }\left(x_{2}\right)=\mu_{\delta \to x_{1}}\left(x_{1}\right)$. Based on the above analysis, we can achieve the node message for *t*th iteration as follows:

(1) Calculate $b_{app}\left(x_{2}\right)$ with $b_{sp}\left(x_{2}\right)\propto p\left(y|Hx_{2},\gamma_{w}^{-1}I\right)p\left(x_{2}|r_{2t},\gamma_{2k}^{-1}I\right)$, then

(15) $${\hat{x}}_{2}={\left(\gamma_{w}H^{H}H+\gamma_{2t}I\right)}^{-1}\left(\gamma_{w}H^{H}y+\gamma_{2t}r_{2t}\right),$$

(16) $$\beta_{2t}^{-1}=\gamma_{2t}\mathrm{var}\left[x_{2}|r_{2t},\gamma_{2t}\right]=\frac{\gamma_{2t}\mathrm{Trace}\left({\left(\gamma_{w}H^{H}H+\gamma_{2t}I\right)}^{-1}\right)}{n}.$$

(2) Calculate $b_{app}\left(x_{1}\right)$ with $b_{app}\left(x_{1}\right)\propto p\left(x_{1}\right)p\left(x_{1}|r_{1t},\gamma_{1t}^{-1}I\right)$, then one key step is to define the prior $p(x_{1})$. Without any assumptions, the prior $p\left(x_{1}\right)$ is unknown; therefore, the shrinkage function $\eta \left(\cdot \right)$ and the divergence $\langle \eta^{{}^{\prime }}\left(r_{k},\gamma_{k}\right)\rangle$ are unknown. The problem is solved in the next subsection.

(3) Calculate the edge message $\mu_{x_{1}\to \delta }\left(x_{1}\right)$ with $\mu_{x_{1}\to \delta }\left(x_{1}\right)\propto \frac{b_{app}\left(x_{1}\right)}{\mu_{\delta \to x_{1}}\left(x_{1}\right)}\propto \frac{\mathrm{CN}\left(x_{1};{\hat{x}}_{1t},\beta_{1t}^{-1}I\right)}{\mathrm{CN}\left(x_{1};r_{1t},\gamma_{1t}^{-1}I\right)}$, then its mean and precision are given by $r_{2t}=\frac{\beta_{1t}{\hat{x}}_{1t}-\gamma_{1t}r_{1t}}{\beta_{1t}-\gamma_{1t}}$ and $\gamma_{2t}=\beta_{1t}-\gamma_{1t}$.

With the forward-and-back message passing, the iterations are converged until the true belief is approximated. The non-loopy factor graph in Figure 2 provides interpretable message scheduling with ease of implementation.

### 3.2. Prior-Aided VAMP Detection

Considering the explicit definition of the sparsity of OFDM-IM symbols in the frequency domain, it can be predicted that the posterior estimation of symbols based on the optimal MMSE criterion can be achieved. x has sparse characteristics and non-zero symbols are drawn from the constellation S. For each element in x, the prior of *x* is given by

(17) $$p\left(x\right)=\frac{\rho }{\left|S\right|}\sum_{s\in S}\delta \left(x-s\right)+\left(1-\rho \right)\delta \left(x\right),$$

where $\rho =\frac{k}{n}$ is the signal sparsity and $\left|S\right|$ is the size of constellation map S. The constellation point *s* has normalized power, i.e., $\frac{\sum_{s\in S}{\left|s\right|}^{2}}{\left|S\right|}=1$.

As shown in step (2), the marginal belief $b_{sp}\left(x\right)\propto \mu_{\delta \to x}\left(x\right)\mu_{p1\to x}\left(x\right)$, which is given by $b_{sp}\left(x\right)\overset{\Delta }{=}\frac{p\left(x\right)p\left(r|x\right)}{\int p\left(x\right)p\left(r|x\right)dx}$, where $p\left(r|x\right)\propto \mathrm{CN}\left(x;\hat{r},v^{r}\right)$ with mean $\hat{r}$ and variance $v^{r}$. The MMSE denosing function is element-wise given by $\hat{x}=E\left[x|b_{sp}\left(x\right)\right]=\frac{\int xp\left(r|x\right)p\left(x\right)dx}{\int p\left(r|x\right)p\left(x\right)dx}$. Expand the denominator as

(18) $$\begin{array}{cc} \int p\left(r|x\right)p\left(x\right)dx & =\mathrm{CN}\left(x;\hat{r},v^{r}\right)\left[\frac{\rho }{\left|S\right|}\sum_{s\in S}\delta \left(x-s\right)+\left(1-\rho \right)\delta \left(x\right)\right]\\ \; & =\frac{\rho }{\left|S\right|}\sum_{s\in S}\frac{\gamma_{w}}{\pi }\exp\left\{-\gamma_{w}{\left|\hat{r}-s\right|}^{2}\right\}+\frac{\gamma_{w}\left(1-\rho \right)}{\pi }\exp\left(-\gamma_{w}{\left|\hat{r}\right|}^{2}\right). \end{array}$$

The numerator is given by

(19) $$\begin{array}{cc} \int xp\left(r|x\right)p\left(x\right)dx & =\int x\mathrm{CN}\left(x;\hat{r},v^{r}\right)\left[\frac{\rho }{\left|S\right|}\sum_{s\in S}\delta \left(x-s\right)+\left(1-\rho \right)\delta \left(x\right)\right]dx\\ \; & =\frac{\rho }{\left|S\right|}\sum_{s\in S}\frac{s\gamma_{w}}{\pi }\exp\left\{-\gamma_{w}{\left|s-\hat{r}\right|}^{2}\right\}. \end{array}$$

The derivative of MMSE denoiser is required to be calculated, which needs the key term $\mathrm{cov}\left[x|b_{sp}\left(x\right)\right]=E\left[x^{2}|b_{sp}\left(x\right)\right]-{\hat{x}}^{2}$. $E\left[x^{2}|b_{sp}\left(x\right)\right]=\frac{\int x^{2}p\left(r|x\right)p\left(x\right)dx}{\int p\left(r|x\right)p\left(x\right)dx}$, where the numerator is given by

(20) $$\begin{array}{cc} \int x^{2}p\left(r|x\right)p\left(x\right)dx & =\int {\left|x\right|}^{2}\mathrm{CN}\left(x;\hat{r},v^{r}\right)\left[\frac{\rho }{\left|S\right|}\sum_{s\in S}\delta \left(x-s\right)+\left(1-\rho \right)\delta \left(x\right)\right]dx\\ \; & =\frac{\rho }{\left|S\right|}\sum_{s\in S}\frac{\gamma_{w}{\left|s\right|}^{2}}{\pi }\exp\left\{-\gamma_{w}{\left|s-\hat{r}\right|}^{2}\right\}. \end{array}$$

With the equation from (18) to (20), the MMSE denoiser and its divergence are achieved, which are utilized to replace step 5 and step 6 in Algorithm 1.

### 3.3. Modification for Invalid SAPs and Constellation Detection

After $\hat{x}$ each group is estimated, the invalid SAPs appear like the case in LLR detection. Instead of discarding the non-existent SAPs as wrong results, modifications are required to reduce the performance loss. Firstly, we sort the power amplitudes of $\hat{x}$ in an ascending order and obtain the indices sequence $\tilde{I}=\left\{i_{0},i_{1},\cdots ,i_{n-1}\right\}$, then the non-zero index group $I=\left\{i_{0},\cdots ,i_{k-1}\right\}$ is guaranteed by the *k* indices with the largest values in $\tilde{I}$. The step is important especially for low SNR cases due to the noise effects greatly blur the boundaries between noise and signal subspace.

However, the member I may be out of the scope of J. To minimize the effects of error detection, the value in the index group is checked to choose the closest one in J. The choice criterion is to minimize the Euclidean distance between I with the Table space, i.e., $\underset{c=1,...,\left|J\right|}{\arg\min}{∥I-J_{c}∥}_{2}^{2}$. It is worth noting that symbols $\hat{x}={\left\{{\hat{x}}_{j}\right\}}_{j=i_{0}}^{i_{k-1}}$ are simultaneously equalized and they have been located on the decision region of the constellation symbols. They are easily recovered using one-step ML symbol detection given by

(21) $${\tilde{x}}_{j}=\underset{s_{q}\in S}{\arg\min}{∥{\hat{x}}_{j}-s_{q}∥}_{2}^{2},j=i_{0},\cdots ,i_{k}.$$

Besides the VAMP with SVD transform in Algorithm 1, here we present the whole process considering the message passing of Figure 2, and the VAMP with LMMSE step is shown in Algorithm 2.
**Algorithm 2** Vector AMP-based OFDM-IM detection (LMMSE Version)**Input:** Received signal of each group $y\in C^{n\times 1}$, measurement matrix $H\in C^{n\times n}$ , Maximum iteration number *T*, denoising function $\eta \left(\cdot \right)$, noise precision $\gamma_{w}$, activated subcarrier number *k***Output:** Recovered index data I and equalized symbols $\tilde{x}$  1:Initialization: t=0, $r_{0}$, $\gamma_{0}\geq 0$  2:**while** t<T **do**  3:      ${\hat{x}}_{1t}=\eta \left(r_{1t},\gamma_{1t}\right)$  4:      $\alpha_{1t}=\langle \eta^{{}^{\prime }}\left(r_{1t},\gamma_{1t}\right)\rangle$  5:      β1t=γ1tα1t  6:      $\gamma_{2t}=\beta_{1t}-\gamma_{1t}$  7:      $r_{2t}=\frac{\beta_{1t}{\hat{x}}_{1t}-\gamma_{1t}r_{1k}}{\gamma_{2t}}$  8:      ${\hat{x}}_{2t}={\left(\gamma_{w}H^{H}H+\gamma_{2t}I\right)}^{-1}\left(\gamma_{w}H^{H}y+r_{2t}\gamma_{2t}\right)$  9:      $\alpha_{2t}=\frac{\gamma_{2t}}{m}*\mathrm{trace}\left[{\left(\gamma_{w}H^{H}H+\gamma_{2t}I\right)}^{-1}\right]$10:      $\beta_{2t}=\frac{\gamma_{2t}}{\alpha_{2t}}$11:      $\gamma_{1,t+1}=\beta_{2t}-\gamma_{2t}$12:      r1,t+1=β2tx^2t−γ2tr2tβ2tx^2t−γ2tr2tγ1,t+1γ1,t+113:**end while**14:Sort $\hat{x}$ as $\tilde{I}=sort\left(\hat{x}{,}^{\prime }ascend^{\prime }\right)$ and estimated indices $I=\tilde{I}\left(n-k+1:n\right)$15:**if** I∉J **then**16:      Update I as $J_{c}$ with the minimum ED in J17:**end if**18:${\tilde{x}}_{j}=\underset{s_{q}\in S}{\arg\min}{∥{\hat{x}}_{j}-s_{q}∥}_{2}^{2},j\in I$

## 4. Numerical Simulations

The numeral experiments are executed through a simulated UWA channel. The results are analyzed to show the advantages of the proposed method. The benchmarks include IM-AMP [9], IM-MMSE-AMP [7], IM-LLR and IM-ML [1] and the proposed detector. The OFDM-IM system parameters are defined in Table 1 unless otherwise stated.

The simulated UWA CIRs are generated from the time-varying UWA channel model [18]. The multipath channel includes *L* = 15 paths and the inter-arrival time follows an exponential distribution with a mean value of 1 ms. The average channel delay spread is 15 ms. The amplitudes of paths follow Rayleigh distribution and the average power of paths decreases exponentially with total power decay 20 dB. The model generates duplicated paths which are merged together. The CFO is randomly generated $\epsilon \in \left[-\frac{\Delta f}{2},\frac{\Delta f}{2}\right]$. For coherent communication systems, accurate CSI is the precondition to guarantee the reliability of the communication links. By exploiting the sparse characteristics of UWA channels, the orthogonal matching pursuit (OMP) method is used in this paper [19].

### 4.1. Complexity Analysis

**Figure 3:**
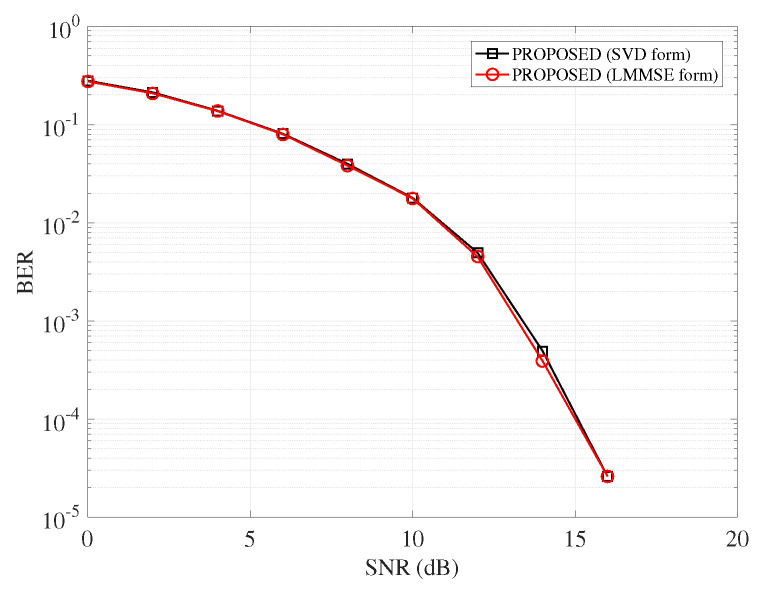
BER performance of the proposed detector with different implementation forms.

The signal detection is operated group by group and the implementation complexity of the mentioned detectors for each group is shown in Table 2. AMP is a low-complexity framework. For AMP-based detectors, the complexity majorly lies in the matrix multiplication operation. For each group, the complexity is $O(n^{2})$. The proposed detector has two types, i.e., the SVD form and the LMMSE form, and the results of the two versions are shown in Figure 3. In Figure 3, both forms of the proposed detector show similar performance. The LMMSE form has matrix inversion operation, and the complexity is $O(n^{3})$ per group. For the SVD form, H is decomposed by SVD transformation and the complexity is O(nR), where *R* is the rank of H. LLR detector works for each subcarrier with the LLR ratio calculation for each constellation point, so the complexity is O(nM) per group. The optimal ML detector requires searching the space of index patterns and constellation maps, so its computation complexity exponentially increased with the index table number as well as the modulation order.

### 4.2. Residual CFO Effects

**Figure 4:**
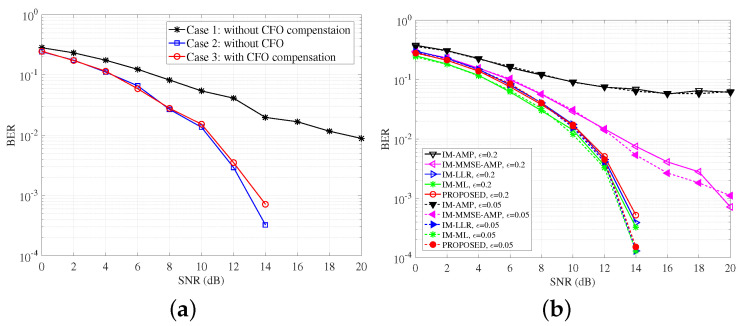
BER comparisons of detectors with different modulation methods. (**a**) BER of ML detector with different residual CFO effects when CFO = 0.2; (**b**) BER comparisons of detectors with CFO compensations when CFO = [0.05 0.2].

Before the investigations on the performance of detectors, CFO effects are first analyzed. To verify the CFO compensation results, Figure 4a presents the BER of the ML detector in three cases when CFO = 0.2. Case 1 considers CFO effects but has no CFO compensation step. Case 2 does not incorporate the CFO effects, which perform as the benchmark. Case 3 incorporates CFO effects as well as the compensation step. From Figure 4, we can see the BER of case 3 is very approximated with the one of case 2, which proves that the CFO compensation method in Section 2 is effective, which greatly reduces the performance losses due to the ICI. At BER = $10^{-2}$, the performance of case 3 is about 7 dB better than case 1.

Figure 4b studies the BER performance of detectors with the CFO compensation in the presence of different CFOs, i.e., $\epsilon =\left[0.05,0.2\right]$. As shown in Figure 4b, besides the ML and LLR detectors, the proposed detector presents robustness to various CFO effects. However, the IM-AMP fails in both cases even considering CFO mitigation. The proposed detector performs better than the IM-MMSE-AMP over the whole SNR range. The results prove that the VAMP framework in the proposed detector is less sensitive to the noise from the UWA channel, such as the channel estimation and ICI. Moreover, the prior-aided denoising function can exploit symbol sparsity and achieve approximated BER compared with the ML and LLR.

### 4.3. BER Performance with Channel Uncertainty

**Figure 5:**
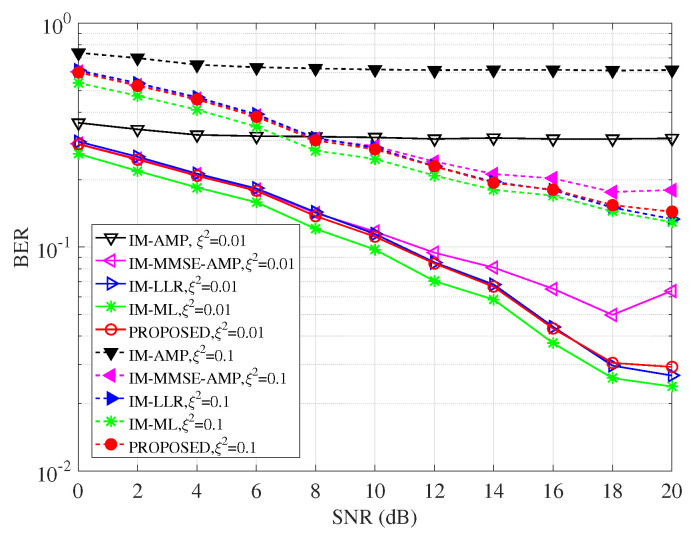
Channel error effects on BER comparisons of detectors when $\xi^{2}$ = [0.01 0.1].

In real applications, the UWA channel cannot be guaranteed in advance. CSI is the prerequisite for data detection. Figure 5 studies the effects of channel uncertainty $\xi^{2}$ on the BER performance of detectors. The CSI uncertainty is incorporated according to $\hat{h}=h+e$, where h and $\hat{h}$ are the true channel vector and estimated channel vectors, respectively. The channel error $e∼\mathrm{CN}\left(0,\xi^{2}I\right)$ with the error variance $\xi^{2}$. Figure 5 considers two CSI conditions with $\xi^{2}=\left[0.01,0.1\right]$. In Figure 5, the larger $\xi^{2}$ causes severe performance losses for detectors. The IM-AMP is too fragile for channel deviation due to channel estimated errors, and it almost loses efficacy due to the non-Gaussianity of the UWA channel matrix. The IM-MMSE-AMP improves the performance and the gains are generated due to the consideration of the symbol prior. The proposed detector performs better than the IM-MMSE-AMP, which both consider the symbol prior but the VAMP framework achieves more gains. The main reasons lie in that the scalar message passing of the VAMP framework has more accurate posterior estimation and robustness to the measurement matrix. Furthermore, the proposed detector performs most approximately with the LLR and the ML. In the case $\xi^{2}=0.01$ and BER $=10^{-2}$, the proposed detector works slightly worse than the ML with a performance gap of about 1dB and the gap with the LLR is within 0.3 dB. In the case $\xi^{2}=0.1$, all detectors are challenged; the proposed detector performs better than the AMP-based benchmarks. The results show that the novel shrinkage function and scalar message passing are effective in recovering the symbol and robust to the measurement matrix with time-varying UWA channel components. It is worthy noting that we use the OMP-based channel estimation method. Actually, the OMP-based method easily achieves an error floor in real UWA channel conditions as in work [17]. To further improve the system performance, a more accurate channel estimation method deserves to be explored, which is not investigated in this paper.

### 4.4. BER Performance with Different System Configuration

**Figure 6:**
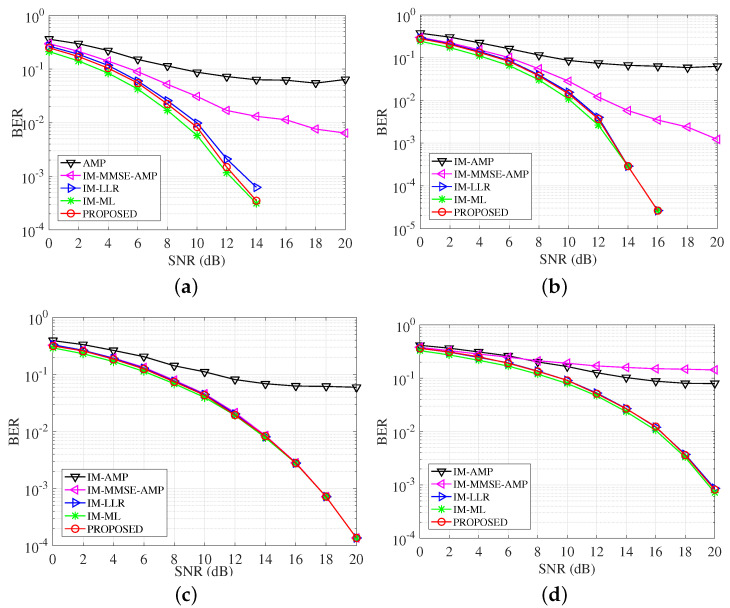
BER comparisons of detectors with different modulation methods. (**a**) BPSK; (**b**) QPSK; (**c**) 8PSK; (**d**) 16QAM.

Figure 6 studies the BER performance of detectors with different constellation modulation, i.e., BPSK, QPSK, 8PSK, and 16QAM. Combined with the setting $\left\{n=4,k=2\right\}$, the data rates of these cases are $\left\{1,1.5,2,2.5\right\}$ bit/s/Hz. In the four subfigures, we can see the IM-AMP has the worst performance. The incorporation of symbol sparsity improves the performance of IM-MMSE-AMP. The IM-MMSE-AMP has relatively good performance in the first three cases, but it diverges in the 16QAM case. The proposed detector outperforms the AMP-based detectors in all considered scenarios and approximates most with the IM-ML along with IM-LLR. In the whole SNR range, the gaps among the three detectors, i.e., the proposed, the IM-LLR and the IM-ML, are within 1dB. As the modulation order increases, the detection is harder due to the decreasing signal spatial distance. Additionally, in our proposed method, the constellation signals are equalized jointly. The better BER performance of our proposed VAMP-based detector proves better index detection results. The results show that the VAMP framework is more robust than the AMP ones when confronting diverse modulation methods.

**Figure 7:**
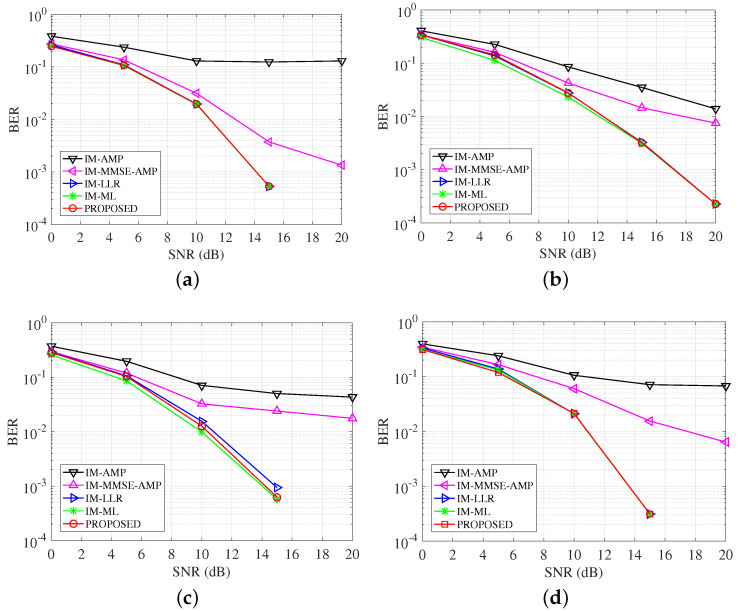
BER comparisons of detectors with different subcarrier allocations. (**a**) (4, 3, 4); (**b**) (8, 3, 4); (**c**) (8, 2, 2); (**d**) (8, 4, 2).

Figure 7 studies the performance of detectors with different combinations of SAPs and constellation points. These cases have the SE $\left\{2,1.375,0.75,1.25\right\}$ bit/s/Hz. In Figure 7, the proposed detector achieves effective results, which are approximated with the ML and the LLR over the whole SNR range. When BER $=10^{-3}$, the proposed detector is almost matched with the IM-ML, and the gain is more than 5 dB compared with IM-MMSE-AMP. The IM-AMP almost works worse due to the sensitivity to the SAPs as well as the errors. The IM-MMSE-AMP presents unstable performance especially in (8, 2, 2), it arrives at its error floor at SNR = 10 dB. OFDM-IM provides us the flexibility to design different systems with desirable SE, where the proposed detector can serve as a low-complexity and effective implementation.

### 4.5. Performance over Different UWA Channel

**Figure 8:**
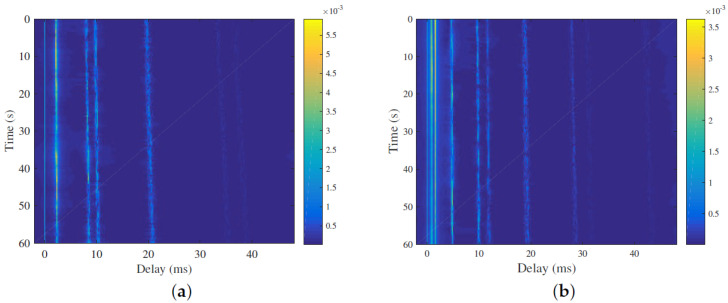
Channel impulse response with different setting parameters. (**a**) Channel 1; (**b**) Channel 2.

**Figure 9:**
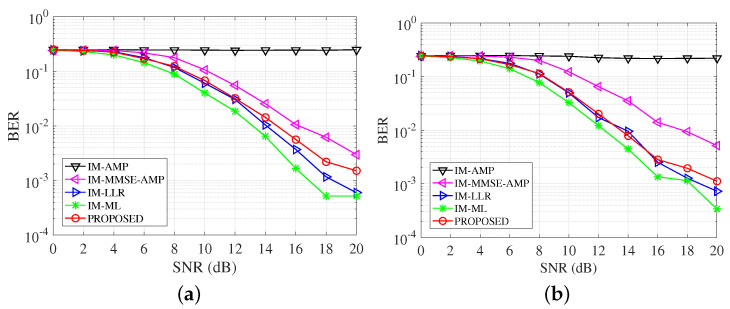
BER comparisons of detectors over different UWA channels. (**a**) BER over channel 1; (**b**) BER over channel 2.

Besides the path-based channel model in the above experiments, the statistical UWA channel model is developed in [20]. This channel model considers detailed ocean physical parameters and deployment conditions. Through setting different deployment parameters as shown in Table 3, the channel impulse responses are shown in Figure 8. These channels present different sparse structures. Pass signals over these channels and CS-based channel estimation is utilized. The BER performance comparisons are shown in Figure 9. From the figure, we can see the proposed detector achieves better BER results compared with the conventional AMP methods. ML has the best BER results over all test channels. Over channel 1, considering the case when BER = $3\times 10^{-3}$, the performance gap between the proposed detector and the LLR is about 1 dB, which is better than the conventional AMP-based detectors with 3 dB gains. Over channel 2, the proposed detector has approximate BER results with the LLR detectors and achieves 4 dB gains compared with the IM-MMSE-AMP detector at BER = $10^{-2}$.

**Figure 10:**
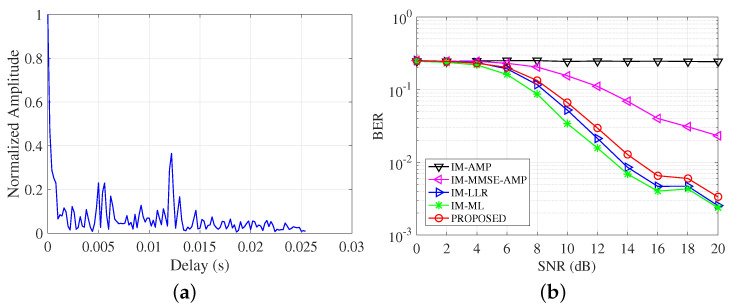
Real channel and BER comparisons of detectors. (**a**) Real channel from Wuyuan Bay; (**b**) BER over Wuyuan Bay channel.

We test the performance over the real-test channel in Wuyuan Bay, Xiamen, China. The channels are sensed and collected by the transceivers at a distance of 1000 m. The transmitter and the receivers are deployed about 4m below the surface. One example of a channel is shown in Figure 10a. From the channel impulse response, we can see the signals are reflected by the sea surface and the bottom. Pass the OFDM-IM signals over the real channel and add different Gaussian noise with a defined SNR level, the BER results are shown in Figure 10b. From the result, we can see the proposed detector keeps its superior performance compared with conventional AMP-based detectors, and most approximates the LLR and ML detectors.

## 5. Conclusions

This paper studies the low-complexity detection for UWA OFDM-IM communication systems. Firstly, we formulate the index detection and the symbol equalization problem into the Bayesian estimation framework. Instead of a well-known AMP framework, the VAMP method with non-loopy FG is utilized to solve the problem. Secondly, considering the symbol sparsity inherently in the OFDM-IM data frame, the prior-aided shrinkage function is derived to achieve better denoising results. For the avoidance of non-existent index detection results, a trick is utilized, which involves the detected erroneous ones into the member of the look-up table. The experimental results show that the proposed detector has reduced complexity, and is robust and effective over different subcarrier allocations and modulation configurations. The proposed method approximates the ML detector, which proves that it can be a competitive alternative for UWA communication applications.

## Figures and Tables

**Table 1:** Parameters for UWA OFDM-IM system.

Bandwidth *B*	5 kHz	No. Subcarrier *N*	1024	Modulation S	QPSK
Subcarrier spacing Δf	4.88 Hz	No. Group *G*	256	Sampling Freq $f_{s}$	100 kHz
CP duration $T_{cp}$	51.2 ms	No. Active subcarrier *k*	2	Center Freq $f_{c}$	12.5 kHz

**Table 2:** Complexity comparison.

AMP	MMSE-AMP	VAMP (SVD/LMMSE)	LLR	ML
$O(n^{2})$	$O(n^{2})$	O(nR)/ $O(n^{3})$	O(nM)	$O\left(2^{\left\lfloor \log_{2}C\left(\begin{array}{c} n\\ k \end{array}\right)\right\rfloor }M^{k}\right)$

**Table 3:** UWA channel parameters.

Parameters	Channel 1	Channel 2
Spreading factor	1.7	1.7
Sound speed in water (m/s)	1500	1500
Sound speed in bottom (m/s)	1200	1200
Surface variance (m${}^{2}$)	0.0125	0.0125
Bottom variance (m${}^{2}$)	0.00625	0.00625
Number of intra-paths	20	20
Mean of intra-path amplitudes	0.025	0.025
Variance of intra-path amplitudes	0.000001	0.000001
Distance (km)	1.5	3
Height of transmitter (m)	45	58
Height of receiver (m)	60	59
Depth of water (m)	100	103

## Data Availability

The data presented in this study are available on request from the corresponding author.
